# Pancreas Transplantation Outcome Predictions—PTOP: A Risk Prediction Tool for Pancreas and Pancreas-Kidney Transplants Based on a European Cohort

**DOI:** 10.1097/TXD.0000000000001632

**Published:** 2024-05-15

**Authors:** Gregor Miller, Donna P. Ankerst, Michael W. Kattan, Norbert Hüser, Felix Stocker, Serge Vogelaar, Milou van Bruchem, Volker Assfalg

**Affiliations:** 1 Department of Surgery, Technical University of Munich (TUM), TUM School of Medicine and Health, TUM – Munich Transplant Center, Klinikum rechts der Isar, Munich, Germany.; 2 Technical University of Munich (TUM), TUM School of Computation, Information and Technology, Garching, Germany.; 3 Core Facility Statistical Consulting, Helmholtz Munich, Neuherberg, Germany.; 4 Department of Quantitative Health Sciences, Cleveland Clinic, Cleveland, OH.; 5 Eurotransplant International Foundation, Leiden, The Netherlands.

## Abstract

**Background.:**

For patients with complicated type 1 diabetes having, for example, hypoglycemia unawareness and end-stage renal disease because of diabetic nephropathy, combined pancreas and kidney transplantation (PKT) is the therapy of choice. However, the shortage of available grafts and complex impact of risk factors call for individualized, impartial predictions of PKT and pancreas transplantation (PT) outcomes to support physicians in graft acceptance decisions.

**Methods.:**

Based on a large European cohort with 3060 PKT and PT performed between 2006 and 2021, the 3 primary patient outcomes time to patient mortality, pancreas graft loss, and kidney graft loss were visualized using Kaplan-Meier survival curves. Multivariable Cox proportional hazards models were developed for 5- and 10-y prediction of outcomes based on 26 risk factors.

**Results.:**

Risk factors associated with increased mortality included previous kidney transplants, rescue allocations, longer waiting times, and simultaneous transplants of other organs. Increased pancreas graft loss was positively associated with higher recipient body mass index and donor age and negatively associated with simultaneous transplants of kidneys and other organs. Donor age was also associated with increased kidney graft losses. The multivariable Cox models reported median C-index values were 63% for patient mortality, 62% for pancreas loss, and 55% for kidney loss.

**Conclusions.:**

This study provides an online risk tool at https://riskcalc.org/ptop for individual 5- and 10-y post-PKT and PT patient outcomes based on parameters available at the time of graft offer to support critical organ acceptance decisions and encourage external validation in independent populations.

## Graphical abstract

**Figure:**
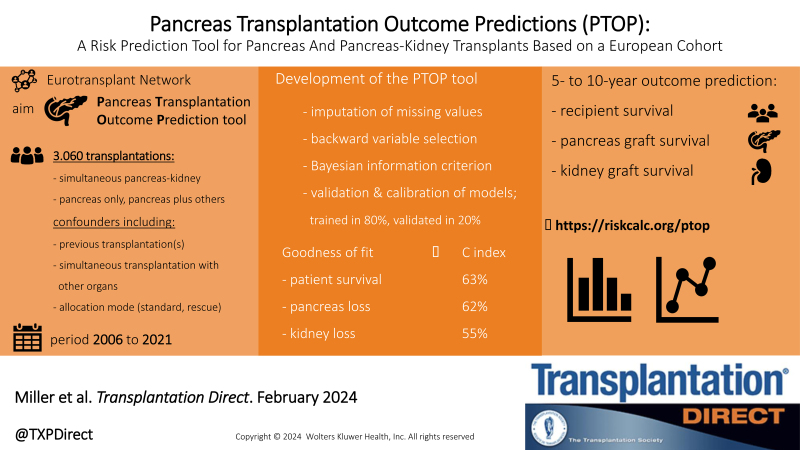

Pancreas-only transplantation (PT) and combined pancreas-kidney transplantation (PKT) are the therapies of choice for suitable patients with type 1 diabetes mellitus having serious complications, such as end-stage renal disease and hypoglycemia unawareness, requiring rescue treatment. In contrast to intense medical treatment of diabetes after kidney transplantation, preferably from a living donor, PKT offers superior metabolic control, long-term insulin independence, alleviation of secondary diabetic complications, improved quality of life, increased long-term survival, and freedom from dialysis.^1-3^ Furthermore, pancreas transplants play an increasingly important role in type 2 diabetes. In a 2018–2019 study of US adults waiting for pancreas transplants, the proportion of type 2 diabetes patients increased from 14.2% to 17.0% in just 1 y, confirming trends from previous years.^4^

Targeted prioritization within Eurotransplant (ET), the largest European international organ allocation organization, ensures that waiting times for PKT in recipients with diabetes are crucially shorter than for kidney-only transplantations.

Despite the outcome benefits of pancreas transplants and increasing diabetes prevalence, their number has declined in recent years in multiple regions, particularly in the United States and Europe. Suggested reasons include continued pancreas graft shortages because of low donation rates, demographic changes comprising increasing age and accumulation of comorbidities, and unawareness of positive outcomes.^5^ In the ET organ allocation system for Austria, Belgium, Croatia, Germany, Hungary, Luxembourg, the Netherlands, and Slovenia, a mean number of 335 candidates were waiting for a PKT between 2012 and 2022. However, a median number of 142 PKT (96–175) were performed, 299 (range, 199–337) recipients were put on the waiting list, 32 candidates (9.6%; range, 18–50) died, and another 52 candidates (15.5%; range, 35–76) were removed from the waiting list per year, respectively. It must be assumed, that most of the patients removed also died in the further course although these data are not collected at ET.

Within the ET pancreas allocation system (EPAS), only 16% of available pancreases were transplanted in 2021.^6^ Previous studies attributed refusals primarily to donor-related factors, including donor age, long stays in intensive care units, resuscitation, or trauma. However, because there is no evidence for many of the stated reasons and refused organs are often transplanted by other centers, refutes at least some of these reasons.^7^

ET may initiate expedited rescue allocation modes to avoid the loss of potentially transplantable grafts. Accordingly, 30% of the transplanted pancreas were rescue-allocated, which is the highest rate among all organs allocated within ET.^6^

Pancreas transplant risk factors previously shown to be associated with posttransplant recipient survival include recipient age, hemodialysis status pretransplant, sensitization at transplantation time, donor age, transplant donor type, duration of dialysis, angina/coronary artery disease, type of exocrine drainage and pancreas donor risk index (PDRI).^8-10^ Risk factors associated with pancreas graft survival included recipient age, sex, body mass index (BMI), transplant donor type, donor age, cold ischemia time, PDRI, retransplantation, and center PT volume.^8,9,11,12^ In addition to patient and pancreas survival, kidney survival is often studied as a relevant outcome because the pancreas and kidney are frequently transplanted simultaneously. Risk factors associated with kidney graft survival include recipient age, sex, BMI, race, donor age, donor/recipient sex pairing, center PT volume, duration of dialysis, angina/coronary artery disease, type of exocrine drainage, and PDRI.^8,12^

The preprocurement pancreas allocation suitability score (P-PASS) and PDRI are often used in practice for outcome prognosis.^13^ The P-PASS is based on donor factors such as age, BMI, intensive care unit stay, cardiac arrest, serum sodium, amylase, lipase, and inotropic therapy. Points are assigned for specified intervals of risk factors or combinations, yielding totals between 9 and 27 points, with lower values indicating better acceptance. However, the P-PASS, developed to identify suitable donors, is incapable of predicting pancreas graft survival, whereas a score of >17 was associated with early pancreas loss.^14^ The P-PASS is indicated by default in the donor report of all potential pancreas donors within ET. The PDRI was created on the basis of data from the US transplant network and uses the donor factors sex, age, race, BMI, cause of death, serum creatinine, height, donation after circulatory death status, cold ischemia time, and transplant type (PKT, pancreas only, or pancreas after kidney). The PDRI was intended to predict the risk of graft failure at 1 y and thus indicate organ quality. Although there is evidence of significant predictive power for the PDRI, validation studies have overall yielded inconsistent predictive utilities of both PDRI and P-PASS.^14-18^

A donor-, graft-, and recipient-specific model for predicting transplant outcomes enables compatibility assessment of any donor organ, allowing for a more substantiated assessment of therapeutic options and greater confidence and efficiency in allocating and accepting the limited supply of available organs. Multivariable survival models have been developed for other organs, including kidney,^19^ liver,^20^ heart,^21^ and lung.^22^

In ET, pancreas grafts of donors aged between 5 and 60 y as well as with a BMI of <30 kg/m^2^ are offered within EPAS. Donors not fulfilling these criteria are considered expanded criteria donors, and their grafts are directed to candidates with special urgency.^23^ Furthermore, transplant candidates are selected on the basis of the required type of transplant, donor age, donor BMI, and ABO blood group. EPAS ranks potential recipients by a scoring system that considers time on the waiting list, donor/recipient distance to minimize cold ischemia time, and international exchange balances. If the standard allocation is not successful, rescue allocation is started.^23^ Rescue allocations include recipient-oriented extended allocations and center-oriented rescue allocations.

Based on a large contemporary European data set of >3000 transplants from the ET system, this study investigated independent donor-, recipient-, and transplant-specific risk factors to develop a patient survival and both pancreas and kidney graft survival probability prediction tool for long-term 5- and 10-y outcomes of PKT and PT, respectively. This tool was internally validated and posted online to facilitate independent external validation and complement decision-making in graft acceptance or refusal.

## MATERIALS AND METHODS

All pancreas transplants from deceased donors performed between 2006 and 2021 in the ET network were considered for inclusion in the study. Eight entries showing inconsistencies were removed, yielding 3060 transplants from 2933 unique recipients for analysis. Transplants were considered to be independent. Ten cases where kidneys, along with other organs, were simultaneously transplanted were excluded from kidney survival outcome analyses because of missing kidney status.

Time from transplant to patient death, pancreas graft loss and kidney graft loss were analyzed for association with donor risk factors, including age, sex, BMI, glucose, creatinine, potassium, sodium, lipase, an indicator of cardiac death or brain death, and smoking status, and recipient risk factors, including, age, sex, BMI, panel-reactive antibodies, waiting time, indicators for previous pancreas transplants, previous kidney transplants and diabetes, the donor/recipient cytomegalovirus IgG-pairing, P-PASS value, cold ischemia time of the pancreas graft, an indicator of standard or rescue allocation, and an indicator of favorable matching of HLAs. The latter was assumed favorable if there was at least 1 HLA-DR and at least 1 HLA-A or 1 HLA-B match; otherwise, the match was considered unfavorable. In the case of kidney graft survival, cold ischemia time of the kidney graft was also included. For pancreas graft and patient survival, indicators of whether a kidney or any other organ was simultaneously transplanted were also considered.

Cox proportional hazards (CPH) models were used for all 3 time-to-event outcomes. Imputation was considered to account for missing values in the risk factors and repeatedly sampled cross-validation was used for model selection. The CPH model with stepwise risk factor selection using the Bayesian information criterion was selected as the modeling approach based on the C-index values and number of selected risk factors produced in the repeatedly sampled cross-validation (**Figure S1, SDC,** http://links.lww.com/TXD/A650); see Appendix 1 (**SDC,** http://links.lww.com/TXD/A650) for details of the model selection approach.

Calibration curves were calculated on the basis of bootstrapping of the full data set. Statistical analyses were performed using the 2-sided 0.05 level of significance, if not otherwise stated, in the R statistical software version 4.2.1 with packages survival, rms, hdnom, randomForestSRC, mboost, survivalmodels, mice, and pec.^24-32^ Analyses were performed after approval by ET authorities (approval code 22005PAC22).

## RESULTS

**FIGURE 1.**
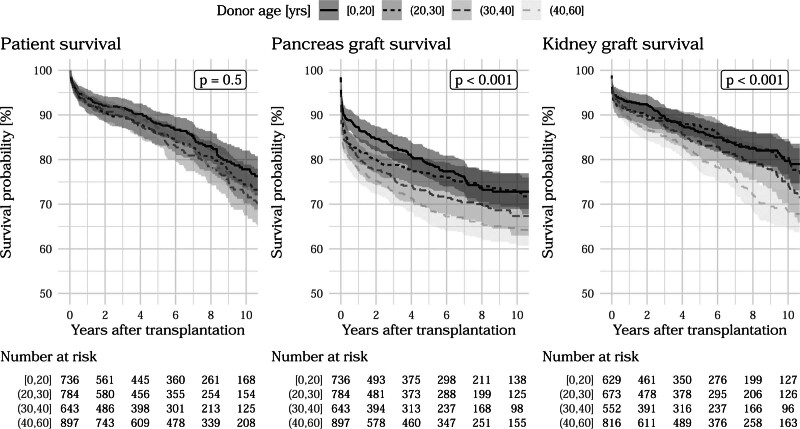
Kaplan-Meier curves of outcomes with respect to donor age with at-risk table and univariable *P* values from likelihood ratio tests of respective Cox proportional hazards model.

**FIGURE 2.**
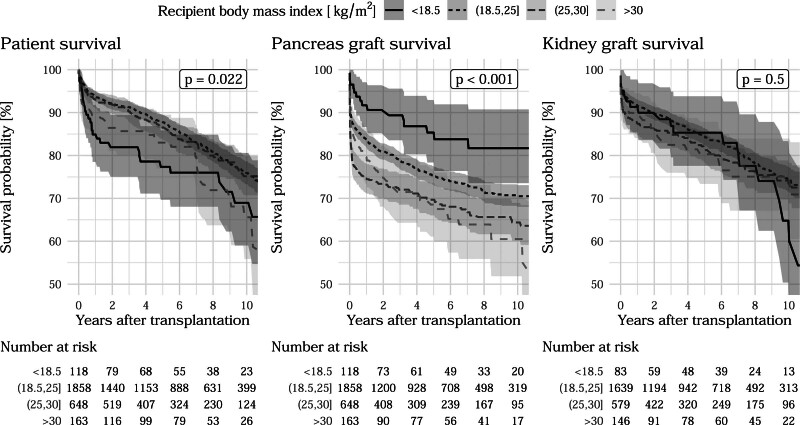
Kaplan-Meier curves of outcomes with respect to recipient body mass index with at-risk table and univariable *P* values from likelihood ratio tests of respective Cox proportional hazards model.

Median follow-up times for censored patient mortality, pancreas loss, and kidney loss were 6.0, 5.8, and 6.0 y, respectively. Among 3060 pancreas grafts, 1895 (62%) were transplanted in Germany, 12% in Austria and 12% in the Netherlands. Patient characteristics in Table 1 show that 92% of the PTs were the first transplantation for recipients and 7.5% were the second. PT after kidney transplantation accounted for 10% of the cases, whereas kidney transplantation was performed simultaneously in 88% of cases. Univariable Cox regression analyses in Table 1 show that previous PKTs were related with inferior recipient survival and pancreas survival. Simultaneous kidney transplants were associated with increased patient survival and pancreas graft survival with hazard ratios (HRs) of 0.51 and 0.46, respectively (both *P* < 0.001). Higher P-PASS values were associated with increased risks of pancreatic and renal graft loss (HR: 1.04 and 1.06, *P* = 0.023 and 0.003). Older donor age was also associated with higher risk of pancreas and kidney loss (HR: 1.01 and 1.02, both *P* < 0.001). Figures 1 and 2 show univariable Kaplan-Meier curves differentiated by donor age and recipient BMI categories, *P* values, and the number of at-risk patients up to 10 y posttransplant. Previous observations that pancreas graft failure often occurred soon after the operation were confirmed, whereas patient mortality and kidney graft losses progressed much steadier over time. An overview of patient and PKT/PT characteristics and univariable associations is given in Table 1.

**TABLE 1.** Transplant characteristics and univariable associations in Cox proportional hazards models with the 3 outcomes regarding patient survival, pancreas graft loss, and kidney graft loss

		Mortality			Pancreas loss			Kidney loss		
Characteristic	n (%)/median (IQR)	HR	95% CI	*P*	HR	95% CI	*P*	HR	95% CI	*P*
Male recipient	1846 (60%)	1.07	0.91-1.27	0.4	1.06	0.92-1.23	0.4	0.90	0.75-1.08	0.2
Male donor	1666 (54%)	0.85	0.72-1.00	0.049	0.94	0.82-1.09	0.4	0.80	0.67-0.95	0.014
Previous pancreas transplant	259 (8.5%)	1.50	1.16-1.94	0.003	1.91	1.56-2.34	<0.001	1.14	0.71-1.82	0.6
Previous kidney transplant	311 (10%)	1.49	1.17-1.89	0.002	1.80	1.48-2.19	<0.001	1.40	0.96-2.04	0.095
Diabetes	2942 (97%)	0.27	0.20-0.36	<0.001	2.99	1.49-6.00	<0.001	0.55	0.23-1.32	0.2
Unknown	18									
Donor cardiac death	92 (3.0%)	0.87	0.43-1.74	0.7	0.86	0.52-1.41	0.5	1.04	0.55-1.94	>0.9
Donor smoking	1080 (41%)	1.09	0.92-1.30	0.3	1.19	1.02-1.39	0.026	1.15	0.95-1.40	0.15
Unknown	421									
CMV-IgG donor (D)/recipient (R) pairing				0.14			0.2			0.4
D^+^/R^+^	577 (26%)	–	–		–	–		–	–	
D^–^/R^+^	510 (23%)	1.36	1.04-1.79		1.03	0.81-1.30		1.22	0.92-1.62	
D^–^/R^–^	611 (27%)	1.15	0.87-1.50		1.08	0.86-1.35		1.00	0.75-1.33	
D^+^/R^–^	536 (24%)	1.08	0.81-1.43		1.27	1.01-1.59		1.16	0.88-1.55	
Unknown	826									
Favorable HLA match^*a*^	1037 (34%)	0.92	0.77-1.10	0.4	1.01	0.87-1.17	0.9	1.10	0.91-1.32	0.3
Unknown	3									
Rescue allocation	724 (24%)	1.17	0.96-1.42	0.14	1.20	1.02-1.41	0.029	1.19	0.97-1.46	0.10
Simultaneous transplant: kidney	2680 (88%)	0.51	0.41-0.63	<0.001	0.46	0.38--0.54	<0.001			
Simultaneous transplant: other organs	93 (3.0%)	5.03	3.77-6.71	<0.001	0.29	0.13-0.64	<0.001			
Recipient age, y	43 (37–50)	1.04	1.03-1.05	<0.001	1.00	0.99-1.01	0.8	1.01	1.00-1.02	0.2
Recipient BMI, kg/m^2^	24.0 (21.0–26.0)	1.01	0.98-1.03	0.6	1.05	1.03-1.07	<0.001	1.01	0.99-1.04	0.4
Unknown	273									
Recipient PRA	0.0 (0.0–0.0)	1.01	1.00-1.01	0.056	1.00	1.00-1.01	0.5	1.01	1.00-1.01	0.2
Unknown	175									
Waiting time, y	1.38 (0.64–2.23)	1.07	1.02-1.13	0.017	0.97	0.92-1.02	0.2	0.97	0.91-1.04	0.4
Donor age, y	31 (21–42)	1.00	1.00-1.01	0.2	1.01	1.01-1.02	<0.001	1.02	1.01-1.02	<0.001
Donor BMI, kg/m^2^	23.0 (21.0–25.0)	1.00	0.97-1.02	0.8	1.03	1.01-1.05	0.013	1.00	0.97-1.03	>0.9
Unknown	1									
Donor glucose, mmol/L	7.50 (6.10–9.00)	1.00	0.99-1.00	0.2	1.00	1.00-1.01	0.032	1.00	1.00-1.01	0.5
Unknown	135									
Donor creatinine, μmol/L	65 (53–84)	1.00	1.00-1.00	0.2	1.00	1.00-1.00	0.4	1.00	1.00-1.00	0.3
Unknown	5									
Donor potassium (K^+^), mmol/L	4.00 (3.70–4.40)	0.99	0.94-1.03	0.4	1.00	0.97-1.02	0.6	0.99	0.94-1.03	0.4
Unknown	22									
Donor sodium (Na^+^), mmol/L	147 (142–152)	1.00	0.99-1.01	0.9	1.00	0.99-1.01	0.8	1.00	0.99-1.01	0.7
Unknown	7									
Donor lipase, μmol/s/L	0.40 (0.25–0.87)	1.00	0.96-1.06	0.9	1.00	0.96-1.05	0.9	0.98	0.92-1.04	0.5
Unknown	414									
P-PASS	15.00 (14.00–17.00)	1.01	0.98-1.05	0.5	1.04	1.01-1.07	0.023	1.06	1.02-1.10	0.003
Unknown	732									
Pancreas CIT, h	9.78 (7.90–11.80)	1.02	0.99-1.05	0.3	1.05	1.02-1.08	<0.001	1.02	0.99-1.05	0.3
Unknown	456									
Kidney CIT, h	10.9 (8.8–13.1)	1.00	0.98-1.03	0.8	1.01	0.99-1.04	0.4	0.99	0.96-1.02	0.6
Unknown	934									

The reference category for donor cardiac death is donor brain death, and for rescue allocation, standard allocation.

^*a*^Favorable HLA match: at least 1 HLA-DR and at least 1 HLA-A or 1 HLA-B match.

BMI, body mass index; CI, confidence interval; CIT, cold ischemia time; CMV, cytomegalovirus; HR, hazard ratio; IQR, interquartile range; P-PASS, preprocurement pancreas allocation suitability score; PRA, panel-reactive antileukocyte antibody.

In the multivariable CPH models, factors associated with higher patient mortality included previous kidney transplants, rescue allocations, longer waiting times, and simultaneous transplants of other organs (Table 2). The main effect HR for simultaneous kidney transplants was far <1 (HR: 0.14); however, this was offset by its interaction with recipient age (HR: 1.04), indicating that as age progressed the risk of mortality for patients with simultaneous kidney transplants increased relative to those with no simultaneous kidney transplants.

**TABLE 2.** Multivariable Cox proportional hazards model estimates

	Mortality			Pancreas loss			Kidney loss		
Characteristic	HR	95% CI	*P*	HR	95% CI	*P*	HR	95% CI	*P*
Previous kidney transplant	1.50	1.12-2.00	0.007						
Allocation type: rescue	1.37	1.12-1.68	0.002						
Waiting time, y	1.08	1.03-1.14	0.002						
Simultaneous kidney transplant: recipient age, y	1.04	1.03-1.06	<0.001						
Simultaneous transplant: other organs	6.25	4.16-9.38	<0.001	0.15	0.07-0.35	<0.001			
Simultaneous transplant: kidney	0.14	0.08-0.25	<0.001	0.35	0.30-0.42	<0.001			
Recipient BMI				1.04	1.02-1.06	<0.001			
Donor age				1.01	1.01-1.02	<0.001	1.02	1.01-1.02	<0.001
Previous kidney transplant: recipient BMI							1.02	1.00-1.04	0.019

BMI, body mass index; CI, confidence interval; HR, hazard ratio.

Lower risks for pancreas graft loss were identified in cases with simultaneous transplants of a kidney or other organs (HR: 0.15 and 0.35), in contrast to increased risk for higher recipient BMI and donor age (HR: 1.04 and 1.01). Higher donor age was also associated with a higher risk of kidney loss (HR: 1.02), similar to the increased effect of higher recipient BMI on increased kidney loss risk when a kidney had been transplanted previously (HR: 1.02).

Median C indices from cross-validation were 63% (10% quantile: 60%, 90% quantile: 66%) for patient mortality, 62% (59%–64%) for pancreas graft loss, and 55% (52%–58%) for kidney graft loss. Calibration plots showed satisfactory agreement between predicted and observed risks 10 y posttransplant (**Figure S2, SDC,** http://links.lww.com/TXD/A650).

## DISCUSSION

PT, usually performed as combined PKT, is characterized by low numbers of donors and numerous adverse prognostic factors of both donors and recipients that accumulate over a lifetime. The end impact culminates in high rates of nonacceptance, rescue allocation, and organ discard.^17^ Decision-making in the event of an organ offer is often challenging for those responsible because of both an urgent need for transplantation because of mortality concerns and high delisting rates on the PKT waiting list on the one hand, but potentially harmful donor-, transplant-, or recipient-specific aspects on the other hand. Therefore, an easily accessible prognostic tool is an attractive resource for transplant physicians who need to decide whether to accept or reject an offer because of the expected poor outcome. In particular, in cases where the physician’s subjective assessment would otherwise lead to rejection because of either lack of confidence or bias, an unbiased prognostic tool based on a large database can support the objective evaluation of the offer and result in increased use of acceptable organs instead of categorical rejection.

This study developed models for 3 post PT and PKT outcomes: patient survival, pancreas graft loss, and kidney graft loss. Based on these models, the first online risk calculator for pancreas transplant outcome predictions (PTOP), including predictions for kidney graft loss was created. The PTOP is based on a large European cohort and can provide accurate 5- and 10-y predictions requiring only the input of a small number of items describing transplant properties, all available at the time when the allocation offer is made. This prompt availability of the parameters needed for calculation simplifies the use of this tool in case of a concrete graft offer to support decision-making. Furthermore, it allows for easy demonstration of the impact of distinct risk factors on transplant outcomes either for medical staff training or for patient education. The risk calculator is available online at https://riskcalc.org/ptop.

Comparison of risk factor selection and modeling methods showed that most methods had comparable results in the analyzed context, and the Bayesian information criterion stepwise selection for a CPH model, as one of the more traditional methods, proved useful in creating compact survival models with high prediction capabilities. Risk factors identified in this study included factors whose associations have already been described in previous studies, including donor age and recipient BMI.^8,9,12^ Importantly however, construction of the models is focused on accurate predictions and not on comprehensive association analyses. It should be noted that the mathematical models accounting for all parameters and combinations of parameters and later restriction to a few of them in the final PTOP system lead to superior prediction accuracy compared with adherence to those covariates medically expected to be relevant. Assumingly, a combination of donor- and transplant-specific factors with a negative impact on outcomes is pooled in the parameter “rescue allocation” according to the EPAS algorithm.

The stepwise selection method introduces uncertainty that is not fully accounted for because of model selection biases^33^ but allows for high interpretability. Furthermore, risk factors may have been included as surrogates for multiple other factors. For example, studies on rescue allocation in kidney transplants have shown that despite indicating risk-increasing associations in univariable analyses, these lose significance when other risk factors, such as age, immunological matching, cold ischemia time, and waiting time, are taken into account. One reason for this behavior is that risk allocations are predominantly performed for recipients with worse outcomes because of higher donor age, higher recipient age, worse immunological matching, and longer cold ischemia time.^34^ The present data set shows similarities in PTs, which must be analyzed in future investigations.

Despite decreasing transplant numbers and more medically complex donors over recent years, accuracy for predicting patient survival and both pancreas and kidney graft survival in this study were optimistic, with PTOP allowing for 10-y predictions with favorable C indices compared with previously published prediction systems.^10,35^

The PTOP is distinguished from the existing scores P-PASS and PDRI insofar as apart from consideration of donor-specific or donor-plus transplant-specific parameters; the PTOP includes important outcome-relevant recipient variables. Moreover, the PTOP does not calculate a score for assessment of, for example, 1-y pancreas graft loss but simultaneously determines concrete 5- and 10-y estimates for patient survival, pancreas graft survival, and even kidney graft survival. This provides physicians and other users with easily interpretable and finely graduated assessments with long-term perspective. The additional prediction of kidney graft outcome of the PTOP calculator in PKT is an unprecedented novum in pancreas transplant scores. The PTOP represents a new category of prediction tools for PT that goes beyond existing scores.

### Limitations

Not all risk factors identified in previous studies as having significant associations with outcomes after pancreatic transplantations could be considered in this study because they were not available in the ET database at the time of publication. This includes recipient duration of diabetic disease, precedent major cardiovascular events, donor ethnicity, PDRI, medication, resuscitation, fine-grained donor laboratory results, and center-specific volume of pancreatic transplantations. Enhancing transplant registries in this regard will strengthen future research. Prediction accuracy in non-ET cases may be limited because of the distinct allocation algorithms, such as ET rescue allocation, which may be not fully comparable with other allocation schemes. Thus, validation of PTOP in countries outside the ET area is of essential importance to determine reproducibility in external populations.

## CONCLUSIONS

The universally available PTOP enables unbiased expectations of posttransplant progress and simulations of a potential candidate’s predicted course based on distinct parameters. Although the decision to accept graft offers remains the responsibility of transplant physicians, impartial and individualized predictions will alleviate decision-making processes that would otherwise require complex and subjective consideration of multiple risk factors and their interactions. Utilization of PTOP will help strengthen confidence in the therapeutic advantages of pancreatic transplantations, improve graft allocation efforts, and potentially help dispel persistent dogmas on single parameters in organ offer evaluation.

## ACKNOWLEDGMENTS

The authors thank ET for providing the data and all ET staff members for their support with the database analyses.

## Supplementary Material


