# Supplementary material for: Pancreas Transplantation Outcome Predictions—PTOP: A Risk Prediction Tool for Pancreas and Pancreas-Kidney Transplants Based on a European Cohort
Source: Transplant Direct. 2024 May 15;10(6):e1632. doi: 10.1097/TXD.0000000000001632 (PMC11098189; doi:10.1097/TXD.0000000000001632)

Supplementary Appendix

Model selection strategy

To identify interactions of risk factors needed to be considered for model selection, Cox proportional hazards models (CPH) were fitted for each pairwise combination and each outcome. The models included the two respective main effects and their interaction. If the interaction showed a p-value below 0.001, it was considered for the respective outcomes. Hence, for patient survival, we added the interaction between a simultaneous kidney transplant and previous kidney transplants as well as the interaction between simultaneous kidney transplant and recipient age. For the kidney outcome, we added the interaction between previous kidney transplants and recipient BMI. For pancreas survival, no interactions showed a p-value small enough to be added.

Multivariable Cox regression was used for all outcomes, including interactions where necessary. To determine the best model for the risk prediction of the three outcomes, repeatedly sampled cross-validation (RSCV) with ten iterations and five folds was used, whereby for each iteration, the data were randomly split into five distinct groups. For each fold, one validation dataset consisting of one of the five groups was withheld for testing and one training dataset consisting of the other groups was used for training the model. Imputed versions of the training and validation datasets using multiple imputations by chained equations blinded to outcome were used to account for missing values. All the modeling techniques were trained on the imputed training set and the corresponding C index was calculated based on the validation set. To select

the most suitable modeling approach, C index values over all iterations and folds as well as the number of used risk factors were compared. For the chosen method, the most often selected combination of incorporated risk factors was then selected as the final model for each respective outcome. The prediction methods were the full CPH model using all predictor values, the stepwise selection CPH model with the Akaike information criterion, the stepwise CPH model with the Bayesian information criterion (BIC), the CPH model with an adaptive elastic-net penalty, the random forest model, the model-based boosting CPH model, and the DeepSurv neural network.

Results of model comparison

Comparison of the C index values over all iterations and folds of the RSCV approach to find the most suitable selection and fitting method showed that almost all methods had very similar results, however, there were isolated downward outliers (Supplementary Figure 1). Additionally, these results were compared with non-imputed validation datasets and obtained comparable results for the median, however, the results showed large variances in particular for methods using all covariates. We selected the BIC method for our models because it had some of the highest median C index values for all three outcomes, needed only few risk factors, and thus showed a comparably small variance between the values of the C index.

For the final models, the covariate combinations were used, which had been selected most often by the BIC method. For patient mortality, four combinations of risk factors were selected equally often (12%). We therefore chose the combination of time on the waiting list, indicators of allocation type, previous kidney transplants, simultaneous transplant of other organs, simultaneous transplant of a kidney, and the interaction between simultaneous kidney transplant

and recipient age. With regards to pancreas loss, donor age, recipient BMI, and indicators of simultaneous transplant of other organs and simultaneous transplant of a kidney were the dominant combination with 28%. For kidney loss, the risk factors were donor age and the interaction between previous kidney transplants and recipient BMI. This combination was selected in 42% of the cases. Overall, the selected combinations indicated the difference between the most predictive risk factors for the three outcomes and showed the importance of a differentiated investigation.

Figure S1: Cox proportional hazards (CPH) models

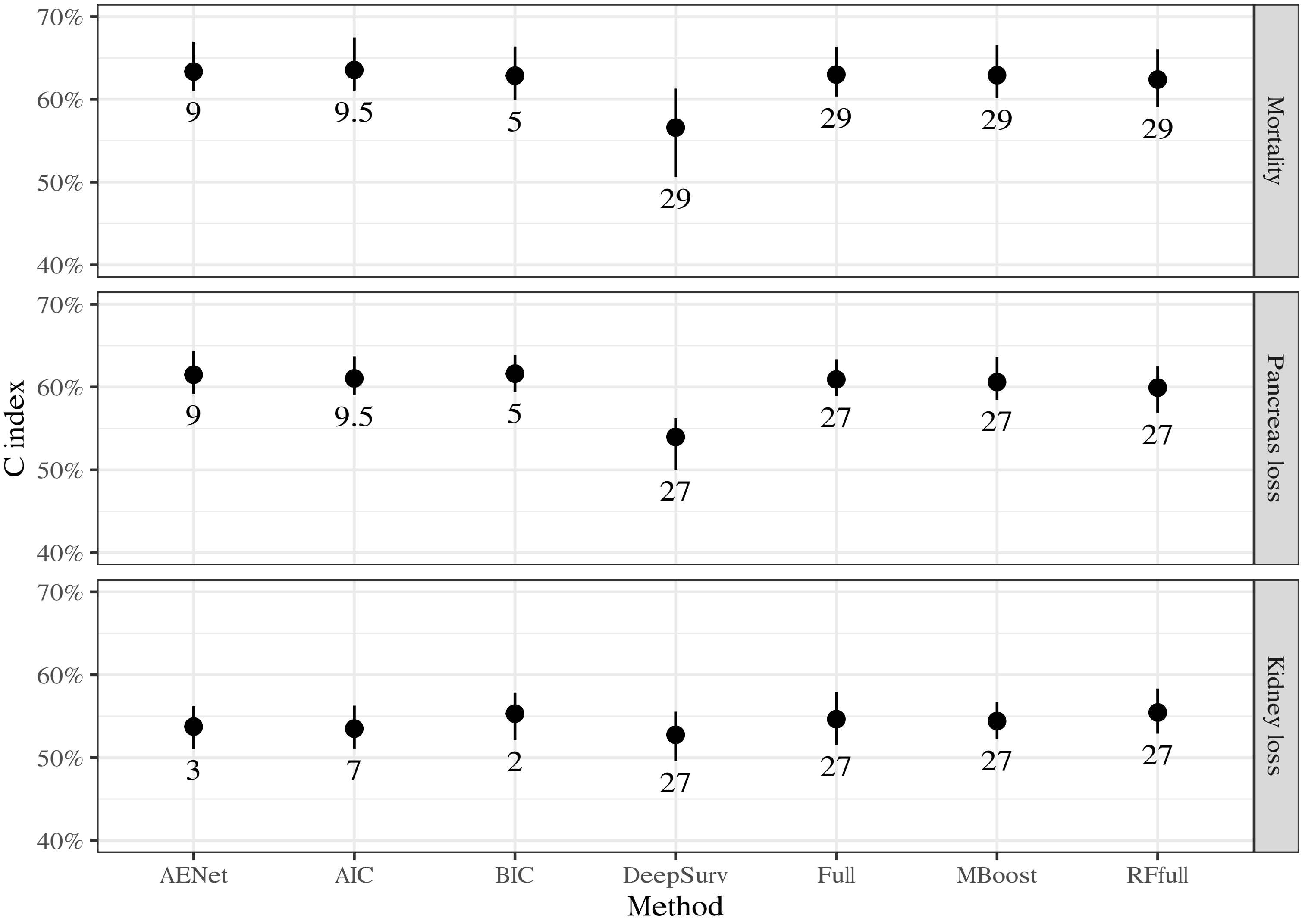

Figure S2: Calibration plots of predicted and observed risks ten years post-transplant

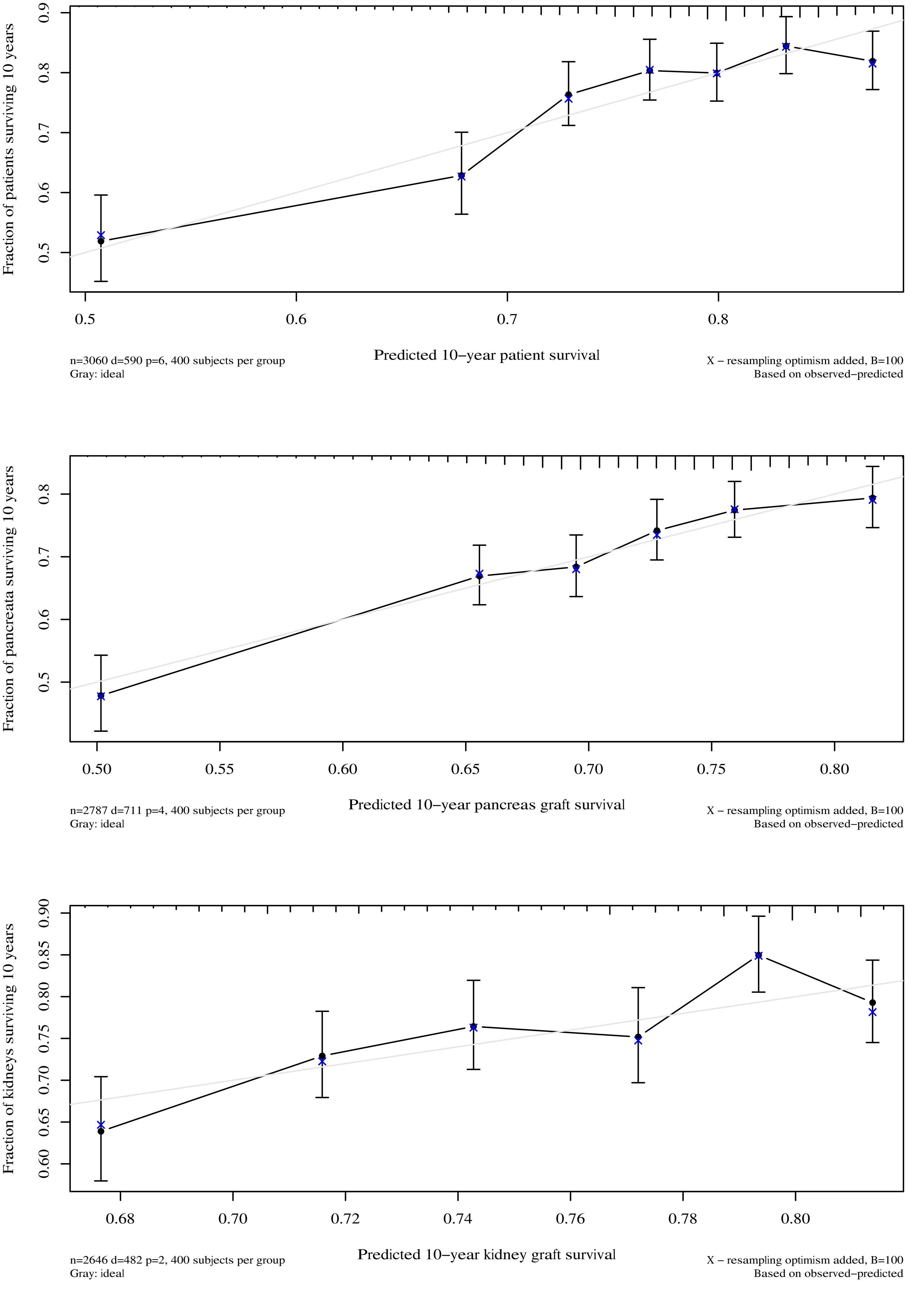

Supplement: Supplementary file 1 [file txd-10-e1632-s001.pdf]
